# In colonic ρ^0^ (rho0) cells reduced mitochondrial function mediates transcriptomic alterations associated with cancer

**DOI:** 10.18632/oncoscience.386

**Published:** 2017-12-27

**Authors:** Harrison M. Penrose, Sandra Heller, Chloe Cable, Hani Nakhoul, Nate Ungerleider, Melody Baddoo, Zachary F. Pursell, Erik K. Flemington, Susan E. Crawford, Suzana D. Savkovic

**Affiliations:** ^1^ Department of Pathology and Laboratory Medicine, Tulane University, New Orleans, LA 70112, USA; ^2^ Department of Biochemistry and Molecular Biology, Tulane University School of Medicine, New Orleans, LA 70112, USA; ^3^ Department of Surgery, NorthShore Research Institute, Affiliate of University of Chicago Pritzker School of Medicine, Evanston, IL 60201, USA

**Keywords:** colon cancer, mitochondria, ρ0 (rho0) cells, transcriptome

## Abstract

**Background:**

Mitochondrial reprogramming has emerged as a hallmark of cancer pathobiology. Although it is believed this reprogramming is essential for cancer cells to thrive, how it supports cancer pathobiology is unclear. We previously generated colonic ρ0 (rho0) cells with reduced mitochondrial energy function and acquired their transcriptional signature. Here, we utilized a bioinformatics approach to identify their changes linked to cancer pathobiology.

**Methods:**

Human colon cancer HCT116 cells, control and ρ0, were used for qPCR. Bioinformatics analysis: GeneCards, Kaplan-Meier Survival, GENT, cBioPortal.

**Results:**

The colonic ρ0 transcriptome was linked with proliferation, DNA replication, survival, tumor morphology, and cancer. Among differentially expressed transcripts, 281 were regulators or biomarkers of human colon cancer especially those with inflammatory microsatellite instability (MSI). We identified and validated novel transcripts in ρ0 cells with altered expression in human colon cancer. Among them DGK1, HTR7, FLRT3, and ZBTB18 co-occurred with established regulators of human colon cancer pathobiology. Also, increased levels of DGKI, FLRT3, ZBTB18, and YPEL1 as well as decreased levels of HTR7, and CALML6 were linked to substantially poorer patient survival.

**Conclusion:**

We identified established and novel regulators in colon cancer pathobiology that are dependent on mitochondrial energy reprogramming and linked to poorer patient survival.

## INTRODUCTION

Metabolic reprogramming has emerged as a new hallmark of cancer progression validating cancer as a metabolic disease [1, 2]. While mechanisms associated with increased glycolysis (Warburg effect) have re- emerged as a focus of cancer research, metabolic reprogramming associated with mitochondria, dynamic organelles known as powerhouses of the cell [3], is not well understood. It is believed that transformed cells would be unable to thrive without mitochondrial reprograming and emerging findings suggest that mitochondrial pleiotropic functions could be critical for cancer progression [2, 4]. As alterations in mitochondrial function could have profound effects on diverse cellular function, understanding the consequences of reprogramming in the pathobiology of cancer is required to define novel mechanisms and reliable targets for new treatment options.

Colon cancer, the second leading cause of cancer-related death in the U.S. (http://seer.cancer.gov/csr/1975_2011/), accounts for more than 694,000 annual deaths worldwide [5]. Colon cancer is driven in part by the microenvironment including imbalances in gut microbiota, inflammation, and obesity [6, 7]. Additional contributing factors in transformed colonic cells include genetic and epigenetic alterations in oncogenes, tumor suppressors, and signaling pathways. Such dysregulation is found with p53, APC, Wnt, KRAS, and PI3K, which consequently favor cellular transformation, proliferation, survival, and subsequent metastasis [8, 9]. Recent findings have revealed that some of these pathways or regulators also have metabolic function. For example, p53 controls various metabolic pathways including glycolysis, lipid metabolism, and mitochondrial function [4, 10]. Limited studies have shown that in some colon cancer cells, the presence of p53 in the mitochondria enhances DNA polymerase function while its deletion leads to disruption of the organelles activity and structure [11, 12], supporting the interconnection between regulators of colon cancer progression and mitochondrial function. Emerging findings have demonstrated colon cancer is associated with mitochondrial DNA deletions, mutations, and migration to the nucleus [3, 13, 14]. However, the role of alterations in mitochondrial function and underlying mechanisms in driving colon cancer are mainly unclear.

Mitochondria, energy producing organelles, utilize their respiration machinery for ATP synthesis (OXPHOS) from the tricarboxylic acid (TCA) cycle and fatty acid β-oxidation [15, 16]. Mitochondria play additional roles in cellular homeostasis by controlling production of reactive oxygen species (ROS), metabolites, and diverse cell signaling including those linked with calcium and cell death [3, 16]. Dysregulation in these mitochondrial functions, such as increased ROS levels, have been shown to foster tumor cell growth and survival [16]. More recent findings show that also dysfunction in mitochondrial biogenesis, networking, signaling, metabolism of fatty acids, and mitophagy are also associated with tumor growth [3, 16, 17], highlighting the role of this organelle in cancer pathobiology. It is plausible that dynamic mitochondrial function influences nuclear gene expression and methylation [18, 19], thus affecting oncogenes, tumor suppressors, and signaling pathways associated with tumor growth. Cells devoid of mitochondrial DNA, known as ρ^0^ (rho^0^), are a reliable model to study cellular function dependent on mitochondria [20], so we generated and characterized human colon cancer ρ^0^ (rho^0^) cells [21]. As ρ^0^ cells are resistant to ROS production and apoptosis [20, 22-24], it is logical to anticipate that the transcriptome of human colonic ρ^0^ cells is mainly dependent on loss of mitochondrial energy function. However, the characteristics of global transcriptomic changes in these cells with reduced mitochondrial energy function in human colon cancer pathobiology is poorly understood and understudied.

Here, by employing next generation RNA sequencing and a bioinformatics approach we identified in colonic ρ^0^ cells transcriptomic changes mediated by reduced mitochondrial energy function. We found substantial similarity between the transcriptomes of ρ^0^ cells and human colon cancer, especially those associated with microsatellite instability (MSI). Also, while a considerable number of established regulators of colon cancer depend on mitochondrial energy function, we also identified novel transcripts whose altered expression was linked to lower patient survival. These findings could facilitate understanding of new mechanisms behind colon cancer pathobiology mediated by mitochondrial energy reprogramming and also establish reliable biomarkers and targets for more efficient diagnosis and treatment options.

## RESULTS AND DISCUSSION

### In colonic cells reduced mitochondrial function leads to altered nuclear gene expression associated with cancer

**Figure 1:**
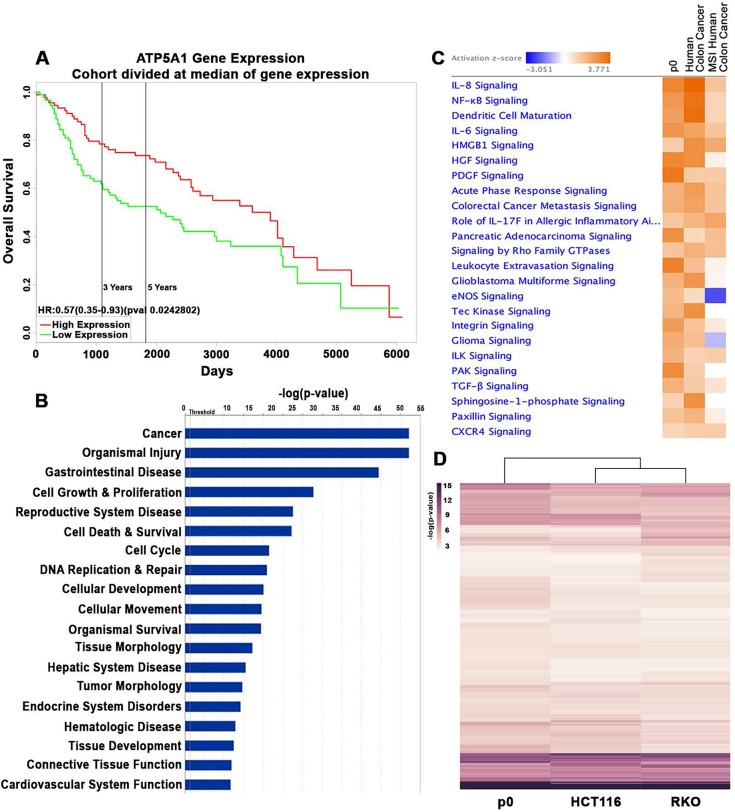
Low mitochondrial energy function promotes cancer signaling A) In human colon cancer, patient survival was plotted for median mRNA expression of mitochondrial ATP5A1 (PROGgeneV2) (p < 0.05). B) Disease and biological functions associated with human colonic ρ^0^ transcriptome (IPA, FDR < 0.05). C) Top canonical pathways activated in human colonic ρ^0^ cells associated with colon cancer with MSI phenotype (TCGA) (IPA, FDR < 0.05). D) Unsupervised hierarchical clustering of canonical pathway activation from transcriptomes of ρ0, HCT116 control, and RKO colon cancer cells (IPA).

Although dysfunction in mitochondrial pleotropic functions have been associated with tumor growth [3, 16, 17], the role of mitochondrial energy reprograming in cancer pathobiology is not well understood. Lower mitochondrial ATP production is associated with colon cancer cell proliferation [25]. We found that decreased ATP synthase subunit ATP5A1, the enzyme responsible for catalyzing ATP production, is linked with considerably reduced colon cancer patient survival (Fig 1A) (p < 0.05). Since this supports a critical role for reduced mitochondrial energy function in colon cancer pathobiology, we analyzed in colonic ρ0 cells with decreased mitochondrial ATP production [21] if differentially expressed (DE) transcripts were associated with human colon cancer. Utilizing Ingenuity Pathway Analysis (IPA), we found that these DE transcripts were linked with cancer and cellular functions associated with cell cycle, proliferation, DNA replication and repair, cellular movement, cell survival, and tumor morphology (Fig 1B) (FDR < 0.05). In addition to gastrointestinal related pathobiology, IPA also revealed that reproductive, endocrine, and cardiovascular system disease phenotypes were affected by reduced mitochondrial function (Fig 1B) (FDR < 0.05). The association of ρ^0^ DE transcripts with cancer of multiple tissues could be due to common mechanisms shared among cancers. Also, it is plausible that reproductive and endocrine pathobiology is a result of the co-dependency of mitochondrial functions with hormone receptor signaling while cardiovascular pathology might be consequence of mitochondrial regulation of calcium levels [4]. Moreover, IPA revealed a considerable similarity between DE transcripts of ρ^0^ cells and human colon cancer (Fig 1C), especially those tumors annotated for MSI, a characteristic defined by ineffective DNA repair leading to hypermutation associated with an inflammatory tumor phenotype [26]. At the signaling level, there was similarity in a number of activated inflammatory pathways including IL-8, IL-6, NFκB, and IL-17 as well as those driving cancer including HGF, PDGF, TGF, PAK, and Integrin signaling (Fig 1C) (FDR < 0.05). The possible mechanisms mediated by mitochondria that lead to colon MSI cancer are unclear. There is a positive correlation between mitochondrial DNA mutation and nuclear MSI in stomach cancer [27]. It could be possible that the MSI phenotype of parental HCT116 cells might contribute to the ρ^0^ transcriptome and similarities shared with MSI human colon cancer, however, their DE transcripts utilized for analysis met a stringent threshold. For example, alteration in expression of established MSI “driver” genes, such as MutL Homolog 1 (MLH1), did not meet the statistical threshold and thus was not a part of ρ^0^ DE transcripts used for analysis. Also, as emerging findings show that mitochondria function could impact inflammation and nuclear DNA replication [21, 26, 28], we speculate, that in colonic ρ0 cells DE associated with MSI phenotype could be the consequence of both inflammation and aberrant DNA replication. Moreover, it is important to consider the colonic ρ^0^ transcriptome might also be driven by parental HCT116 cell mutations in K-ras and β-catenin genes (KRAS, CNNTB1) [29], Thus we performed unsupervised hierarchical clustering of activated canonical pathways from ρ^0^ transcriptomes with parental HCT116 cells and colon cancer RKO cells with wild-type K-ras and β-catenin. We found a considerably higher transcriptional overlap between parental HCT116 and RKO cells than with ρ^0^ cells (IPA) (Fig 1D), suggesting that the ρ^0^ transcriptome is influenced notably more by reduced mitochondrial function than by mutational alterations these cells may carry. Together these data demonstrate that in colon cancer cells, reduced mitochondrial function leads to nuclear transcriptional changes associated with dysregulation of multiple cellular functions leading to inflammatory MSI cancer.

### Reduced mitochondrial function mediates expression of regulators, biomarkers, and novel facilitators of colon cancer

Next, we identified in the colonic ρ^0^ cells transcripts involved in the pathobiology of colon cancer. IPA recognized 281 transcripts that were associated with human colorectal adenocarcinoma including 20 currently recognized as biomarkers for diagnostic purposes (Supplemental 1). Among these transcripts were members of the mucin, matrix metallopeptidase, claudin, fibronectin, ankyrin, vimentin, and cyclin dependent kinase families suggesting mitochondrial dysregulation affects multiple cellular functions. Whether expression of these transcripts is directly or indirectly regulated by mitochondria needs to be determined, our analysis indicates that energy reprograming of this organelle plays a central role in the pathobiology of colon cancer.

**Figure 2:**
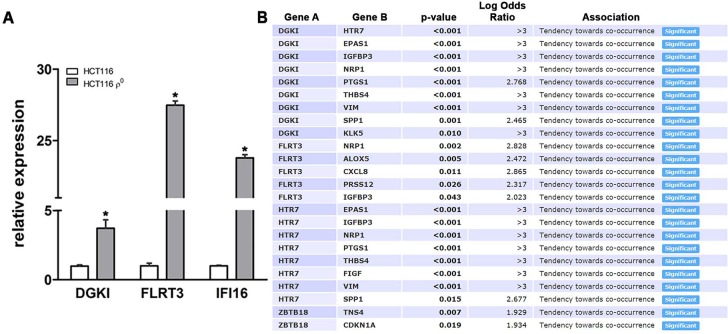
Novel transcripts from colonic ρ^0^ cells are co-expressed with established regulators of colon cancer A) RNA from HCT116 cells, control and ρ^0^, was used for quantitative PCR of selected transcripts: novel (DGKI and FLRT3) and established (IFI16) (n=4, *p < 0.05 relative to control). B) Co-occurrence and mutual exclusivity plots for differentially expressed selected transcripts in ρ^0^ cells and known regulators and biomarkers of colorectal cancer (TCGA, cBioPortal, p < 0.05).

Furthermore, we identified novel transcripts in the colonic ρ^0^ transcriptome that are involved in various cell functions, but have not been recognized in cancer pathobiology (genecards.org). Elevated levels of diacylglycerol kinase iota (DGKI), fibronectin leucine rich transmembrane protein 3 (FLRT3), zinc finger and BTB domain containing 18 (ZBTB18 (or ZNF238)), and yippee like 1 (YPEL1) were observed, while transcripts decreased in expression included 5-hydroxytryptamine receptor 7 (HTR7) and calmodulin like 6 (CALML6). Changes in expression in colonic ρ^0^ cells of selected transcripts, DGKI and FLRT3, were verified by qPCR (Fig 2A). As a positive control of elevated transcripts measured in ρ^0^ cells, Interferon Gamma Inducible Protein 16 (IFI16), known to be involved in colon cancer [30], was confirmed (Fig 2A). Moreover, we found that expression of these transcripts was connected with established drivers of human colon cancer pathobiology as they had 135 co-occurring or mutually exclusive expression patterns, of which 24 co-occurred in a significant pattern (Fig 2B) (p < 0.05). For example, in human colon cancer tissues DGKI, a regulator of lipid signaling, and HTR7, a neurotransmitter receptor, were coordinately expressed with each other and with EPAS1 and prostaglandin-endoperoxide synthase 1 (PTGS1). Expression of FLRT3, involved in cell adhesion and receptor signaling, co-occurred with arachidonate 5-lipoxygenase (ALOX5), and insulin like growth factor binding protein 3 (IGFBP3); ZBTB18, a transcriptional repressor, with cyclin dependent kinase inhibitor 1A (CDKN1A). Together, we showed that mitochondrial function mediated expression of novel transcripts involved in diverse cellular function co-occur with expression of established regulators of colon cancer.

### Expression of novel transcripts dependent on reduced mitochondrial function in human colon cancer tissues is associated with poor survival

**Figure 3:**
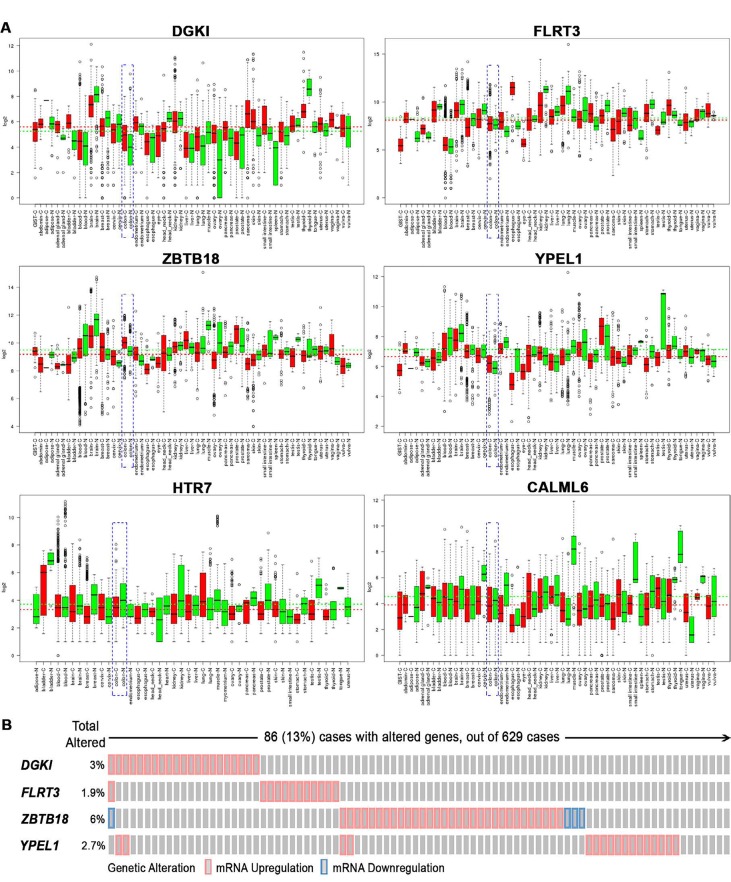
Novel transcripts from colonic ρ^0^ cells have altered expression across human cancer tissues A) Expression of DGKI, FLRT3, ZBTB18 and YPEL1 is increased while expression of HTR7 and CALML6 is decreased in human colon cancer tissues relative to normal across the GENT database. Boxes (red – cancer and green – normal) show the median and 25th and 75th percentiles, whereas dots represent outliers; the horizontal red and green dashed lines represent average expression values for each selected gene across normal and tumor tissues. Colon cancer has been highlighted in the dashed blue box. B) Alterations in expression of the four selected transcripts increased in ρ^0^ cells among human colon cancer tissues. Individual transcripts are represented as rows, whereas individual colon cancer cases are shown as columns (TCGA, cBioPortal).

**Figure 4:**
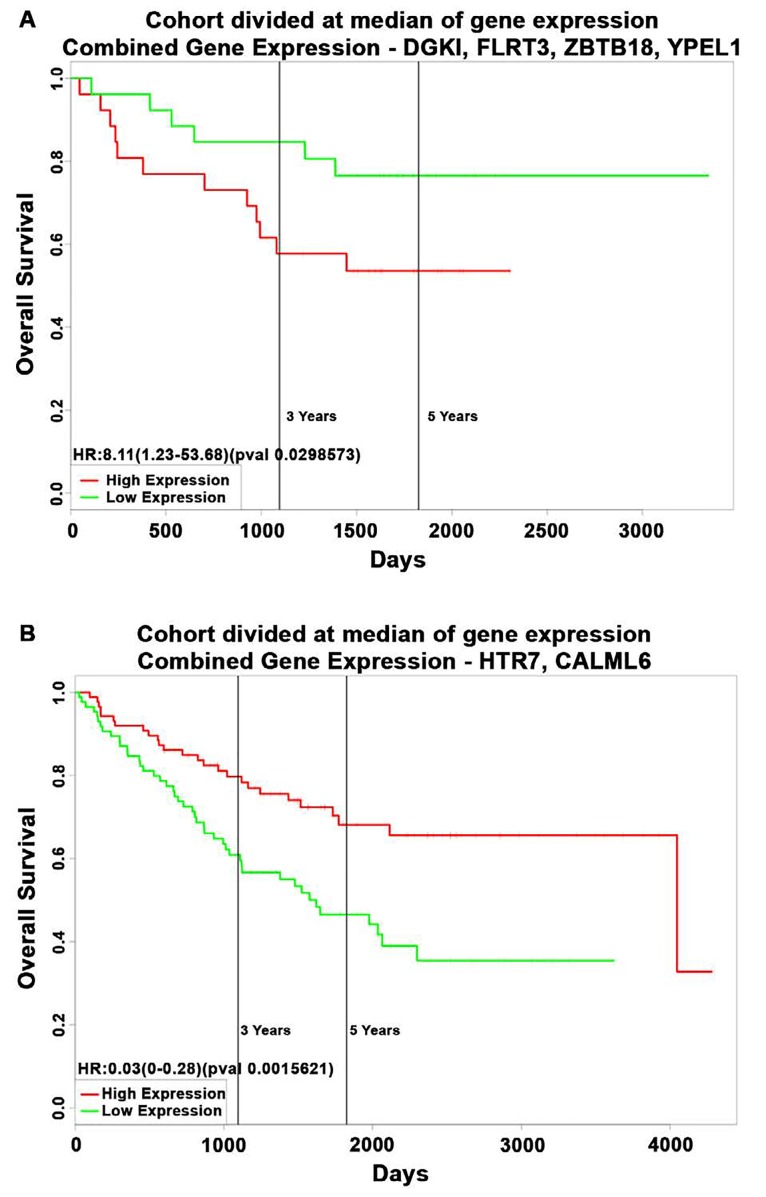
Kaplan-Meier survival estimates of human colon cancer patients with alterations in novel transcripts from colonic ρ^0^ cells (A) Poorer patient survival was linked with increased levels of DGKI, FLRT3, ZBTB18, and YPEL1 expression (PROGgeneV2) (p < 0.05). (B) Poorer patient survival was linked with decreased levels of HTR7 and CALML6 expression (PROGgeneV2) (p < 0.05).

As the function of these six DE transcripts in ρ^0^ cells is unexplored we assessed if their levels were altered in human colon cancer tissues across 2,500 individual patient microarray samples from the GENT database. We found in human colon cancer relative to normal tissues that elevated DGKI, FLRT3, ZBTB18 and YPEL1 transcripts were also increased, while attenuated HTR7 and CALML6 transcripts were lowered (Fig 3A). Their altered expression was also observed in cancers of other digestive tissues such as stomach, liver, and pancreas as well as in reproductive tissues, yet their expression appears to vary based on tissue type. Also, we found that among human colon cancer elevated DGKI, FLRT3, ZBTB18 and YPEL1 transcripts were particularly dysregulated in roughly 13% of patients (Fig 3B). Furthermore, a combined increase in DGKI, FLRT3, ZBTB18, and YPEL1 levels as well as a combined decrease in HTR7 and CALML6 levels were associated with significantly poorer patient survival (Fig 4A,B, p < 0.05). Together, we identified that six novel regulators of diverse cellular function, transcriptionally dependent on mitochondrial activity, are involved in colon cancer pathobiology. Although the mechanisms utilized by these novel regulators in colon cancer pathobiology needs to be determined we hypothesized that they might be directly or indirectly facilitating the disease. DGKl has functions in lipid metabolism and its increase was found in hepatocellular carcinoma [31]. It is plausible that increased DGK1 function creates lipids as building blocks for membranes of highly proliferative cancer cells and lipid signaling could promote inflammation associated with MSI phenotype [32]. Dysregulation of FLRT3 and ZBTB18, as a transmembrane protein and transcriptional repressor, could have broad implications in cancer pathobiology. It is recently reported that ZBTB18 is associated with glioblastoma progression [33]. Furthermore, the role of HTR7, a serotonin receptor, in cancer is largely unknown, yet it has been recently implicated in macrophage activity and inflammation [34], both important in the tumor inflammatory microenvironment [35]. CALML6 regulates calcium levels [36], which was recently suggested as a potentially new target to galvanize tumor suppressors for cancer treatment [37]. Also, regulators of calcium levels are implicated in the inflammatory response as well as cancer signaling [38, 39]. Lastly, YPEL1, a member of the YPEL family, localizes to the centrosome and plays a role in cell division [40], which could support cancer progression, yet the mitochondria-YPEL-centrosome axis needs further understanding. We speculate that decreased YPEL1 levels could lead to aberrant centrosome function thereby driving genomic instability as seen with MSI colon cancer. Moreover, since altered expression patterns of these novel regulators are variable among cancers of different tissues we speculate this could be mediated by influences from the surrounding microenvironment. Specifically, the ability of cancer cells to uptake functional mitochondria from surrounding cells [41] reveals that dynamic mitochondrial reprogramming with cancer pathobiology could be cancer-tissue specific, requiring further exploration of their function in this regard.

Collectively, we found in colonic cells that reduced mitochondrial energy function leads to transcriptomic changes associated with human colon cancer pathobiology, especially those with MSI. We also identified that a substantial number of established regulators linked to colon cancer are dependent on mitochondrial reprograming. Furthermore, we demonstrated novel transcripts with altered expression in human colon cancer tissues correlate with reduced patient survival. Alterations in mitochondrial pleiotropic functions are found to facilitate diverse human diseases including cancer; thereby, becoming a prime area of research in the past decade. This wide spectrum of diseases includes diabetes mellitus, multiple sclerosis, leigh syndrome, neuropathy, mitochondrial myopathy, rheumatoid arthritis, systemic lupus, Inflammatory Bowel Disease, Parkinson's, Alzheimer's and Huntington's diseases [4, 16, 21, 42]. Emerging findings support that reprograming of mitochondrial pleiotropic functions facilitate cancer pathobiology, which could also underlie ethnic differences in tumor diversity and aggressiveness seen in colon cancer [2, 4, 16]. These findings provide insights into novel signaling mechanisms mediated by mitochondrial reprograming favoring cancer pathobiology and provide the potential opportunity for a new therapeutic approach for cancer treatment.

## MATERIAL AND METHODS

### Tissue culture and human colon cancer ρ^0^ (rho0) cells

Human colon cancer HCT116 cells (ATCC) were cultured in McCoy's 5A media (Sigma-Aldrich) supplemented with 10% fetal bovine serum (FBS) (Thermo Fisher Scientific). Human colon cancer HCT116 ρ^0^ (rho0) cells were propagated in media containing 15% FBS, 100µM pyruvate (Thermo Fisher Scientific), 50µg/ml uridine (Sigma-Aldrich), and 2.5µM ethidium bromide and passaged 8-10 times [20]. Loss of mitochondrial DNA was confirmed by PCR as described before [21]. Before experimental procedures, cells were incubated overnight in media without serum.

### RNA isolation, cDNA synthesis, sequencing, clustering, differential expression, and pathway analysis

RNA isolation, cDNA synthesis, sequencing, differential expression testing, and pathway analysis were performed as described before [21, 43]. Sequencing data for this study is available through NCBI's Sequence Read Archive (SRA) with accession number SRP093357. Raw transcriptomic reads from RKO colon cancer cells were obtained from SRA study SRP074476 (accession numbers: SRR3479758, SRR3479759, SRR3479760). After alignment and transcript quantification was performed as referenced above, transcriptomes from ρ0 cells, HCT116 control, and RKO cells were subject to unsupervised hierarchical clustering using Ingenuity Pathway Analysis (Qiagen Inc.). The transcriptional signature of colonic ρ0 cells was compared to expression data derived from human colorectal cancer (CRC) samples (GSE4183, NCBI GEO). For comparison of the colonic ρ0 cell expression signature against expression data from inflammatory human microsatellite instable (MSI) human colon cancers, quantified RNA-seq data was obtained from The Cancer Genome Atlas (TCGA, https://portal.gdc.cancer.gov) [44] via Firebrowse (firebrowse.org).

### qPCR

Total RNA was extracted with QIAzol Lysis Reagent (Qiagen) followed by reverse transcription employing the SuperScript First-Strand Synthesis System (Thermo Fisher Scientific) and Oligo-dT12-18 primers according to the standard protocol [21, 43]. The following primers were used for quantification of cDNA specifically binding FLRT3 (FLRT3-FOR 5′-GGTATTGCGGGTGCAAGATG-3′, FLRT3-REV 5′-GCCATCCCACGAACCTTTTC-3′), DGKI (DGKI- FOR 5′-CAGGTCTCGTACAGGAAAGCA-3′, DGKI REV-5′-ACTCCACTCCAAAGTCGCTC-3′), IFI16 (IFI16-FOR 5′-GAAGTGCCAGCGTAACTCCT-3′, IFI16-REV 5′-ACCTCAAACACCCCATTCACA-3′), and HPRT1 (HPRT1-FOR 5′-GACCAGTCAACAGGGGACAT-3′, HPRT1-REV 5′-AACACTTCGTGGGGTCCTTTTC-3′). Relative levels of mRNA were calculated with HPRT1 as the housekeeping gene using the comparative Ct method.

### Gene function and analysis

Gene function in human health and disease was assessed using GeneCards (www.genecards.org).

### Kaplan-Meier survival plots

Survival analysis of human CRC adenocarcinoma patients for selected genes was accomplished by PROGgeneV2 [45] (http://www.compbio.iupui.edu/ proggene). PROGgeneV2 survival was determined for individual genes or gene signatures from the following cohorts GSE41258, GSE17537, GSE17536. Cohorts were bifurcated using median expression of each gene or gene signature of interest.

### GENT

Gene expression across normal and tumor tissues was accomplished using the GENT database for cancer bioinformatics [46] (http://medical-genomics.kribb.re.kr/GENT/). This database has collected public expression depositories from Gene Expression Omnibus (GEO) and Array Express using the Affymetrix U133 platforms.

### cBioPortal for cancer genomics

The cBioPortal for human cancer genomics (www.cbioportal.org) was employed for generation of OncoPrints and co-expression and mutual exclusivity plots of selected transcripts in human colorectal adenocarcinoma patient samples [47] (TCGA COAD). Selected genes were analyzed using a z-score threshold of ±2, meaning that selected transcripts were significantly altered (up or down) at least 2 standard deviations away from their mean expression in human colon cancer (RNA-seq V2 RSEM; Case Set: All Tumors (629 patients /633 samples)).

### Statistical analysis

Analysis of variance (ANOVA) and Student- Newman-Keuls post-test or Student's unpaired t test were used for statistical analysis employing GraphPad Instat 3 software (GraphPad Software). All data are shown as means ± S.E. for a sequence of experiments and a p value < 0.05 was considered to be statistically significant.

## SUPPLEMENTARY MATERIALS TABLE



## Abbreviations

DGKI: diacylglycerol kinase iota
FLRT3: fibronectin leucine rich transmembrane protein 3
ZBTB18: zinc finger and BTB domain containing 18
YPEL1: yippee like 1
HTR7: 5-hydroxytryptamine receptor
CALML6: calmodulin like 6
CRC: colorectal cancer
MSI: microsatellite instability
GEO: Gene Expression Omnibus
TCGA: The Cancer Genome Atlas
IPA: Ingenuity Pathway Analysis
GENT: Gene Expression across Normal and Tumor tissue
FDR: false discovery rate
