# Supplementary material for: In colonic ρ0 (rho0) cells reduced mitochondrial function mediates transcriptomic alterations associated with cancer
Source: Oncoscience. 2017 Dec 27;4(11-12):189–98. doi: 10.18632/oncoscience.386 (PMC5769983; doi:10.18632/oncoscience.386)
Supplement: Supplementary file 1 [file oncoscience-04-0189-s001.pdf]

SUPPLEMENTARY MATERIALS

Supplemental 1: Differentially expressed transcripts in colonic ρ^0 cells altered in human colorectal adenocarcinoma (fold change >│2│, IPA), *established biomarkers in diagnosis, disease progression, efficacy, prognosis, and response to therapy.

Transcript	Entrez Gene Name	Fold Change
MMP13	matrix metallopeptidase 13	42.80
PMEPA1	prostate transmembrane protein, androgen induced 1	36.24
DCLK1	doublecortin like kinase 1	29.71
*VIM	Vimentin	28.68
FSTL5	follistatin like 5	21.00
TNC	tenascin C	19.88
GLIS3	GLIS family zinc finger 3	16.23
IFI16	interferon gamma inducible protein 16	15.43
DNAH12	dynein axonemal heavy chain 12	13.89
ZEB1	zinc finger E-box binding homeobox 1	12.79
HRNR	hornerin	11.60
SLITRK6	SLIT and NTRK like family member 6	11.07
HMCN1	hemicentin 1	10.81
FLG	filaggrin	10.67
SAMD9L	sterile alpha motif domain containing 9 like	10.65
GRIK2	glutamate ionotropic receptor kainate type subunit 2	10.17
GRM5	glutamate metabotropic receptor 5	9.39
LCN2	lipocalin 2	9.28
LURAP1L	leucine rich adaptor protein 1 like	9.13
MGAM	maltase-glucoamylase	8.94
KCNH8	potassium voltage-gated channel subfamily H member 8	8.70
SLIT2	slit guidance ligand 2	8.69
THBS1	thrombospondin 1	8.68
PRKAA2	protein kinase AMP-activated catalytic subunit alpha 2	8.41
*CXCL8	C-X-C motif chemokine ligand8	8.33
SLC2A3	solute carrier family 2 member 3	8.08
PAPPA2	pappalysin 2	7.99
NEDD9	neural precursor cell expressed, developmentally down-regulated 9	7.96
MUC16	mucin 16, cell surface associated	7.81
COL6A6	collagen type VI alpha 6 chain	7.60
MAST4	microtubule associated serine/threonine kinase family member 4	7.46
DMBT1	deleted in malignant brain tumors 1	7.32

CPEB2	cytoplasmic polyadenylation element binding protein 2	7.19
*NRP1	neuropilin	7.06
FBN2	fibrillin 2	7.03
PKD1L1	polycystin 1 like 1, transient receptor potential channel	6.88
CLDN2	claudin 2	6.75
TG	thyroglobulin	6.71
SLCO1B1	solute carrier organic anion transporter family member 1B1	6.67
CPA4	carboxypeptidase A4	6.57
GBP1	guanylate binding protein 1	6.46
IL7R	interleukin 7 receptor	6.32
MAML2	mastermind like transcriptional coactivator 2	6.26
FAT4	FAT atypical cadherin 4	6.14
FN1	fibronectin 1	6.14
ABCB1	ATP binding cassette subfamily B member 1	5.96
*GDF15	growth differentiation factor 15	5.71
NRG1	neuregulin 1	5.62
SERPINE2	serpin family E member 2	5.52
NRP2	neuropilin 2	5.23
ALDH1A3	aldehyde dehydrogenase 1 family member A3	5.23
*SPP1	secreted phosphoprotein 1	5.23
KIAA0408	KIAA0408	5.16
ALS2CR11	ALS 2 chromosome region candidate 11	5.09
DOCK4	dedicator of cytokinesis 4	5.06
PDE3A	phosphodiesterase 3A	5.04
PCDHGB1	protocadherin gamma subfamily B, 1	5.03
DPF3	double PHD fingers 3	4.95
SCUBE1	signal peptide, CUB domain and EGF like domain 1	4.83
ANK1	ankyrin 1	4.73
MAP1B	microtubule associated protein 1B	4.67
RUNX2	runt related transcription factor 2	4.66
HCLS1	hematopoietic cell-specific Lyn substrate 1	4.60
PLK2	polo like kinase 2	4.48
MSH4	mutS homolog 4	4.46
ADCY10	adenylate cyclase 10, soluble	4.26
KCNMA1	potassium calcium-activated channel subfamily M alpha 1	4.20
PCDHGA9	protocadherin gamma subfamily A, 9	4.15
NEB	nebulin	4.11
SYTL2	synaptotagmin like 2	4.09
CD22	CD22 molecule	4.08
CUBN	cubilin	3.94
PLAU	plasminogen activator, urokinase	3.91

HKDC1	hexokinase domain containing 1	3.90
*CDKN1A	cyclin dependent kinase inhibitor 1A	3.86
*IGFBP3	insulin like growth factor binding protein 3	3.86
HSD17B3	hydroxysteroid 17-beta dehydrogenase 3	3.77
SMPD1	sphingomyelin phosphodiesterase 1	3.68
SCN2A	sodium voltage-gated channel alpha subunit 2	3.48
NWD1	NACHT and WD repeat domain containing 1	3.48
LONRF2	LON peptidase N-terminal domain and ring finger 2	3.46
ARSJ	arylsulfatase family member J	3.44
CYP1B1	cytochrome P450 family 1 subfamily B member 1	3.31
DYSF	dysferlin	3.27
PTPRR	protein tyrosine phosphatase, receptor type R	3.23
COL6A3	collagen type VI alpha 3 chain	3.22
SEMA3A	semaphorin 3A	3.14
LOXL2	lysyl oxidase like 2	3.12
*ALOX5	arachidonate 5-lipoxygenase	3.12
LMO7	LIM domain 7	3.09
MAFA	MAF bZIP transcription factor A	3.06
PCDHGA6	protocadherin gamma subfamily A, 6	3.02
SAMD4A	sterile alpha motif domain containing 4A	3.00
GAB2	GRB2 associated binding protein 2	2.96
ZFY	zinc finger protein, Y-linked	2.95
MECOM	MDS1 and EVI1 complex locus	2.94
ASAP2	ArfGAP with SH3 domain, ankyrin repeat and PH domain 2	2.94
PRSS35	protease, serine 35	2.91
GALNT5	polypeptide N-acetylgalactosaminyltransferase 5	2.89
TLR3	toll like receptor 3	2.87
IMPG2	interphotoreceptor matrix proteoglycan 2	2.87
CNTN1	contactin 1	2.86
ALS2CR12	ALS 2 chromosome region candidate 12	2.84
PIK3R1	phosphoinositide-3-kinase regulatory subunit 1	2.83
MSTN	myostatin	2.80
C1orf168	chromosome 1 open reading frame 168	2.79
TMTC2	transmembrane and tetratricopeptide repeat containing 2	2.79
ARFGEF3	ARFGEF family member 3	2.79
ANTXR2	anthrax toxin receptor 2	2.77
ITGA10	integrin subunit alpha 10	2.77
*TNS4	tensin 4	2.76
SLC16A4	solute carrier family 16 member 4	2.74
ZNF365	zinc finger protein 365	2.73

TOB1	transducer of ERBB2, 1	2.70
PLPPR4	phospholipid phosphatase related 4	2.69
HEG1	heart development protein with EGF like domains 1	2.64
SYNPO2	synaptopodin 2	2.60
NCKAP5	NCK associated protein 5	2.56
*VEGFD	vascular endothelial growth factor D	2.56
RPE65	RPE65, retinoid isomerohydrolase	2.55
CHAC1	ChaC glutathione specific gamma-glutamylcyclotransferase 1	2.52
SLC26A7	solute carrier family 26 member 7	2.52
*THBS4	thrombospondin 4	2.52
LAMA3	laminin subunit alpha 3	2.51
LYG1	lysozyme g1	2.50
GBP3	guanylate binding protein 3	2.50
ABCA12	ATP binding cassette subfamily A member 12	2.50
PTPRZ1	protein tyrosine phosphatase, receptor type Z1	2.49
NR3C2	nuclear receptor subfamily 3 group C member 2	2.49
GLI2	GLI family zinc finger 2	2.48
LRP1B	LDL receptor related protein 1B	2.48
NES	nestin	2.46
*KLK5	kallikrein related peptidase 5	2.45
IGSF1	immunoglobulin superfamily member 1	2.44
TEX14	testis expressed 14, intercellular bridge forming factor	2.43
ST6GALNAC3	ST6 N-acetylgalactosaminide alpha-2,6-sialyltransferase 3	2.41
ATP8B3	ATPase phospholipid transporting 8B3	2.38
CSF2RA	colony stimulating factor 2 receptor alpha subunit	2.38
FAM214B	family with sequence similarity 214 member B	2.37
AXDND1	axonemal dynein light chain domain containing 1	2.34
SOX9	SRY-box 9	2.33
CD58	CD58 molecule	2.32
LRRK1	leucine rich repeat kinase 1	2.31
VEZF1	vascular endothelial zinc finger 1	2.31
PALM2-AKAP2	PALM2-AKAP2 readthrough	2.28
*MDM2	MDM2 proto-oncogene	2.26
SNPH	syntaphilin	2.27
LGALS9	galectin 9	2.25
AGAP6	ArfGAP with GTPase domain, ankyrin repeat PH domain 5	2.25
PTPRM	protein tyrosine phosphatase, receptor type M	2.24
ADAMTS14	ADAM metallopeptidase with thrombospondin type 1 motif 14	2.23
HOXB3	homeobox B3	2.21
NOX5	NADPH oxidase 5	2.20
*PRSS12	protease, serine 12	2.19

PLOD2	procollagen-lysine,2-oxoglutarate 5-dioxygenase 2	2.18
WDR47	WD repeat domain 47	2.18
CLU	clusterin	2.15
NBEA	neurobeachin	2.13
TRIM6	tripartite motif containing 6	2.12
RPS6KA2	ribosomal protein S6 kinase A2	2.12
SMAD3	SMAD family member 3	2.09
C2orf16	chromosome 2 open reading frame 16	2.06
GCLM	glutamate-cysteine ligase modifier subunit	2.05
ATP10D	ATPase phospholipid transporting 10D (putative)	2.03
*EPAS1	endothelial PAS domain protein 1	2.03
TFPI	tissue factor pathway inhibitor	2.02
FAS	Fas cell surface death receptor	2.01
ARFGAP3	ADP ribosylation factor GTPase activating protein 3	2.01
CCNG2	cyclin G2	2.01
SLC46A3	solute carrier family 46 member 3	2.01
CAMK1D	calcium/calmodulin dependent protein kinase ID	2.00
CHEK2	checkpoint kinase 2	-2.00
PASK	PAS domain containing serine/threonine kinase	-2.01
MYC	v-myc avian myelocytomatosis viral oncogene homolog	-2.01
NCAPD3	non-SMC condensin II complex subunit D3	-2.01
*CDKN2A	cyclin dependent kinase inhibitor 2A	-2.02
MED12L	mediator complex subunit 12 like	-2.03
POLE	DNA polymerase epsilon, catalytic subunit	-2.04
ETV4	ETS variant 4	-2.04
KCND3	potassium voltage-gated channel subfamily D member 3	-2.05
GRB14	growth factor receptor bound protein 14	-2.06
AARS2	alanyl-tRNA synthetase 2, mitochondrial	-2.08
ESRP2	epithelial splicing regulatory protein 2	-2.09
*PTGS1	prostaglandin-endoperoxide synthase 1	-2.09
BRIP1	BRCA1 interacting protein C-terminal helicase 1	-2.10
CHTF18	chromosome transmission fidelity factor 18	-2.10
KNTC1	kinetochore associated 1	-2.11
PROM1	prominin 1	-2.11
SCNN1A	sodium channel epithelial 1 alpha subunit	-2.11
ZNF695	zinc finger protein 695	-2.12
PCSK9	proprotein convertase subtilisin/kexin type 9	-2.13
SASS6	SAS-6 centriolar assembly protein	-2.13
RCC2	regulator of chromosome condensation 2	-2.14
TMEM97	transmembrane protein 97	-2.14
RAD54B	RAD54 homolog B (S. cerevisiae)	-2.14

MARVELD3	MARVEL domain containing 3	-2.15
RPS6KA3	ribosomal protein S6 kinase A3	-2.15
C21orf58	chromosome 21 open reading frame 58	-2.16
ZNF114	zinc finger protein 114	-2.17
POLD1	DNA polymerase delta 1, catalytic subunit	-2.18
CARD10	caspase recruitment domain family member 10	-2.18
SLC16A9	solute carrier family 16 member 9	-2.19
CYP24A1	cytochrome P450 family 24 subfamily A member 1	-2.19
TICRR	TOPBP1 interacting checkpoint and replication regulator	-2.20
FANCE	Fanconi anemia complementation group E	-2.20
L3MBTL3	l(3)mbt-like 3 (Drosophila)	-2.22
XRCC2	X-ray repair cross complementing 2	-2.23
CDC6	cell division cycle 6	-2.24
TCF7L2	transcription factor 7 like 2	-2.24
SCLY	selenocysteine lyase	-2.24
AMH	anti-Mullerian hormone	-2.26
LRIG1	leucine rich repeats and immunoglobulin like domains 1	-2.28
POLA1	DNA polymerase alpha 1, catalytic subunit	-2.28
PXDN	peroxidasin	-2.29
EPN3	epsin 3	-2.29
LIG1	DNA ligase 1	-2.29
RAD51AP1	RAD51 associated protein 1	-2.31
CLUH	clustered mitochondria homolog	-2.32
HELLS	helicase, lymphoid-specific	-2.32
NFIA	nuclear factor I A	-2.35
FGD3	FYVE, RhoGEF and PH domain containing 3	-2.36
POLQ	DNA polymerase theta	-2.37
BEND3	BEN domain containing 3	-2.37
AIM1	absent in melanoma 1	-2.37
NR2C2AP	nuclear receptor 2C2 associated protein	-2.38
ATAD2	ATPase family, AAA domain containing 2	-2.40
CR2	complement C3d receptor 2	-2.41
JUNB	JunB proto-oncogene, AP-1 transcription factor subunit	-2.41
FAM111B	family with sequence similarity 111 member B	-2.41
TRAF2	TNF receptor associated factor 2	-2.46
ELFN2	extracellular leucine rich repeat and fibronectin type III domain containing 2	-2.46
EGR1	early growth response 1	-2.47
GINS1	GINS complex subunit 1	-2.48
ROR2	receptor tyrosine kinase like orphan receptor 2	-2.51
KIF7	kinesin family member 7	-2.53

ADAMTS2	ADAM metallopeptidase with thrombospondin type 1 motif 2	-2.56
ICAM5	intercellular adhesion molecule 5	-2.56
*EXO1	exonuclease 1	-2.57
IQGAP2	IQ motif containing GTPase activating protein 2	-2.57
*MSH6	mutS homolog 6	-2.59
LRP3	LDL receptor related protein 3	-2.65
FHDC1	FH2 domain containing 1	-2.69
EOMES	eomesodermin	-2.72
MCM7	minichromosome maintenance complex component 7	-2.75
CYP4F2	cytochrome P450 family 4 subfamily F member 2	-2.76
HAGHL	hydroxyacylglutathione hydrolase-like	-2.78
UNC13A	unc-13 homolog A	-2.79
VWA1	von Willebrand factor A domain containing 1	-2.79
MID1	midline 1	-2.81
ZNF648	zinc finger protein 648	-2.81
JPH1	junctophilin 1	-2.91
POLE2	DNA polymerase epsilon 2, accessory subunit	-2.91
ANK3	ankyrin 3	-2.97
MCM4	minichromosome maintenance complex component 4	-2.98
WASF3	WAS protein family member 3	-2.98
CDCA7	cell division cycle associated 7	-3.00
FABP6	fatty acid binding protein 6	-3.13
MAGI1	membrane associated guanylate kinase, WW and PDZ domain containing 1	-3.20
MCM10	minichromosome maintenance 10 replication initiation factor	-3.21
PON3	paraoxonase 3	-3.33
SPSB4	splA/ryanodine receptor domain and SOCS box containing 4	-3.36
RHOU	ras homolog family member U	-3.41
BLM	Bloom syndrome RecQ like helicase	-3.49
DDX60L	DEAD-box helicase 60-like	-3.54
DAPK1	death associated protein kinase 1	-3.55
SALL4	spalt like transcription factor 4	-3.79
CACNA1D	calcium voltage-gated channel subunit alpha1 D	-3.83
SLC43A3	solute carrier family 43 member 3	-3.93
TBX4	T-box 4	-4.56
LINGO1	leucine rich repeat and Ig domain containing 1	-4.69
KLRG2	killer cell lectin like receptor G2	-4.80
MEF2C	myocyte enhancer factor 2C	-4.85
TMPRSS13	transmembrane protease, serine 13	-5.53
KLHDC9	kelch domain containing 9	-5.92

DEGS2	delta 4-desaturase, sphingolipid 2	-6.17
SLC12A5	solute carrier family 12 member 5	-7.20
PRUNE2	prune homolog 2	-13.46
DMRT1	doublesex and mab-3 related transcription factor 1	-14.40
*TERT	telomerase reverse transcriptase	-16.92
NLRP5	NLR family pyrin domain containing 5	-18.98
KDM4C	lysine demethylase 4C	-47.31
